# Kinetics and mechanisms of flumequine degradation by sulfate radical based AOP in different water samples containing inorganic anions†

**DOI:** 10.1039/d2ra00199c

**Published:** 2022-03-30

**Authors:** Yuanyuan Zhang, Kunling Huang, Yunjie Zhu, Xuan Chen, Min Wei, Kefu Yu

**Affiliations:** School of Marine Sciences, Guangxi Key Laboratory on the Study of Coral Reefs in the South China Sea, Guangxi University Nanning 530004 China 8668370@qq.com; Southern Marine Science and Engineering Guangdong Laboratory (Zhuhai) Nanning 530004 China

## Abstract

Many studies have reported that hydroxyl radical (HO˙) driven advanced oxidation processes (AOPs) could degrade fluoroquinolones (FQs) antibiotics effectively. Compared with HO˙, sulfate radical (SO_4_˙^−^) shows a similar oxidation capacity but a longer half-life. SO_4_˙^−^ could cause chain reactions and resulted in the generation of halogen radicals and carbonate radicals from the main anions in sea water including Cl^−^, Br^−^ and HCO_3_^−^. However, few studies were focused on the degradation of FQs in marine aquaculture water and seawater, as well as the bioaccumulation of transformation products. As a typical member of FQs, flumequine (FLU) was degraded by UV/peroxodisulfate (PDS) AOPs in synthetic fresh water, marine aquaculture water and seawater. The reaction rate constants in the three water samples were 0.0348 min^−1^, 0.0179 min^−1^ and 0.0098 min^−1^, respectively. The reason was attributed to the inhibition of the anions as they could consume SO_4_˙^−^ and initiate the quenching reaction of free radicals. When the pH value increased from 5 to 9, the reaction rate decreased from 0.0197 min^−1^ to 0.0066 min^−1^. The energy difference between HOMO and LUMO of FLU was calculated to be 8.07 eV indicating that FLU was a stable compound. The atoms on quinolone ring of FLU with high negative charge would be more vulnerable to attack by free radicals through electrophilic reactions. Two possible degradation pathways of FLU were inferred according to the degradation products. Preliminary bioaccumulation analysis of transformation products by the EPI suite software proved that the values of log *K*_ow_ and log BCF of the final product P100 were less than those of FLU and the intermediates.

## Abstract 2

Many studies have reported that hydroxyl radical (HO˙) driven advanced oxidation processes (AOPs) could degrade fluoroquinolones (FQs) antibiotics effectively.

## Graphical abstract

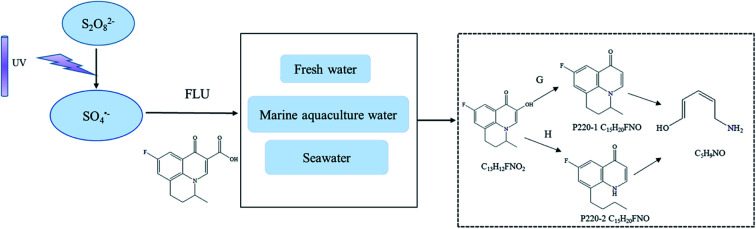

## 1. Introduction

Fluoroquinolones (FQs) antibiotics have been widely used in veterinary medicine, animal husbandry and commercial aquaculture due to their potent antibacterial activity.^1,2^ However, these compounds have been continuously introduced into the environment and caused direct ecological risks impacts.^3^ As their stability and biological resistance, most antibiotics cannot be efficiently degradated by traditional biological water treatment processes.^4,5^ It is necessary to develop an efficient process for FQs removal because of the low degradation efficiencies by the traditional waste water treatments plants.

As a member of FQs group, flumequine (FLU) has been frequently detected in various waterbodies at concentrations ranging from ng L^−1^ to μg L^−1^.^6,7^ The degradations of FLU by different methods have been investigated in the past few years. Lou *et al.* indicated that the flumequine efficiency removal reached 94% by coupling of heterogeneous photocatalysis with ozonation and hydrogen peroxide.^8^ Harrabi *et al.* found that the degradation of FLU was improved by the presence of hydroxyl radicals and sulfate radicals.^9^ A study indicated that the effective removal of flumequine by combined UV/ClO_2_ advanced oxidation processes (AOPs).^10^ Solis *et al.* found that the enhanced elimination of flumequine in the presence of peroxymonosulfate (PMS) and magnetic graphene catalysts.^11^ Feng *et al.* pointed out that multiple additions of Fe(vi) improved the degradation efficiency of FLU, and provided greater degradation than a single addition of Fe(vi).^12^ Zeng *et al.* indicated that the TiO_2_/AN sample exhibited high photocatalytic performance under simulated sunlight irradiation, and 100% removal efficiency of FLU was achieved after 4 hours of illumination.^13^ Ashrafi *et al.* found that the effective removal of FLU from aqueous solution by laccase from *Trametes versicolor*, and the removal rate can reach 77.76% under the optimum conditions.^14^ Many studies have reported that hydroxyl radical (HO˙, *E*_0_ = 2.5–3.1 V, <1 μs) driven advanced oxidation processes (AOPs) such as photocatalytic and Fenton processes could degrade FLU effectively.^15,16^ Compared with HO˙, sulfate radical (SO_4_˙^−^) shows a similar oxidation capacity (*E*_0_ = 1.9–2.8 V) but a longer half-life (30–40 μs).^17–19^ Moreover, the radical precursors peroxodisulfate (PDS) and peroxymonosulfate (PMS) are stable and easily activated.^20^ As a potential alternative for traditional AOPs, SO_4_˙^−^ based AOP has been proven to be effective for the removal of antibiotics.^21,22^

SO_4_˙^−^ is generated through homolysis or heterolysis of the peroxide (O–O) bonds in persulfate anions *via* electron or energy transfer.^23^ There are various methods of activating PS/PMS to produce SO_4_˙^−^, such as ultraviolet (UV) irradiation, heating, ultrasound, transition metal catalysis, nanostructured carbons,alkali activation and carbon based material activation.^24^ Among them, ultraviolet (UV) irradiation is environmentally friendly and commonly used as the activation methods (eqn (1) and 2).^25,26^ At present, UV/PMS has been widely used in degradation of various antibacterial agents in water and wastewater, such as the chloramphenicol,^27^ CIP and AMO,^28^ sulfaquinoxaline,^29^ norfloxacin,^30^ ofloxacin,^31^ ciprofloxacin, metronidazole (MNZ),^32^ sulfamethoxazole (SMX) and sulfamethoxypyridazine (SMP).^33^ The UV/PDS (PMS) process has been reported to be a possible method for removing antibiotics including FLU. For example, Qi *et al.* reported that FLU was able to be degraded by UV/PMS in different water matrices, followed the order of tap water > ultrapure water > river water > secondary clarifier effluent, and the presence of HCO_3_^−^ could inhibited FLU removal, and Cl^−^ concentrations did not significantly affect the reaction. The results showed that UV/PMS is a feasible technology for treating FLU in waters and wastewaters.^34^ On the contrast, Cl^−^ could inhibited FLU removal in our experiments. Therefore, it is necessary to explore the degradation of FLU in synthetic fresh water, marine aquaculture water and seawater and their constituents.

Some studies suggest that the addition of persulfate to water is not pernicious to humans. For example, the effective removal of 2-methylisoborneol and 2-methylisoborneol-producing algae by sulfate radical-based technology in drinking water sources.^35^

Considering that FLU has been directly applied as feed additive in marine aquaculture, the process of FLU degradation may be different as the water was a mixture of seawater and fresh water at ratio of 1 : 2.^36^ SO_4_˙^−^ was reported to be more susceptible to halide scavenging than that of HO˙. Consequently, SO_4_˙^−^ could cause chain reactions and resulted in the generation of halogen radicals and carbonate radicals from the main anions in sea water including Cl^−^, Br^−^ and HCO_3_^−^ (eqn (3)–(7)).^37,38^ Such as HO˙, SO_4_˙^−^ can relatively oxidize all organic matters without selectivity with near-diffusion-limited rate constants. However, the oxidation abilities of halogen radicals (except for Cl˙) to organic matters are selective, for example, they can react faster with certain organic compounds (*e.g.*, alkenes, aromatics, and organothiols) (*k* = 10^6^ to 10^10^ M^−1^ s^−1^) than other organic compounds (*e.g.*, aliphatic molecules) (*k* = 10^3^ to 10^6^ M^−1^ s^−1^).^39^ Therefore, the new second generation radicals may also be responsible for the degradation of FLU due to its complex structure. Our previous studies investigated the degradation of sulfamethoxazole (SMX) and ofloxacin (OFL) by UV/PDS in different water samples.^31,33^ The results indicated that the reaction rates for both SMX and OFL followed the order of synthetic seawater > synthetic marine aquaculture water > freshwater. Anions in seawater showed different effects on SMX and OFL degradation. Therefore, the effects of main anions in seawater on FLU degradation of SO_4_˙^−^ based AOP need to be further investigated. Furthermore, the toxicity evaluation of the degraded products is also very necessary for the food safety of seafood.1
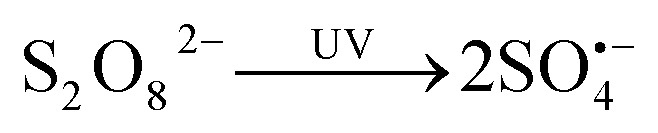
2
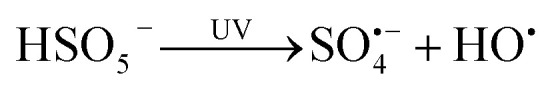

3 SO_4_˙^−^ + Cl^−^ → Cl˙ + SO_4_^2−^*k* = 2.7 × 10^8^ M^−1^ s^−1^

4 Cl˙ + Cl^−^ → Cl_2_˙^−^*k* = 8 × 10^9^ M^−1^ s^−1^

5 Cl˙ + Cl_2_˙^−^ → Cl_2_ + Cl^−^

6 SO_4_˙^−^ + Br^−^ → Br˙ + SO_4_^2−^*k* = 3.5 × 10^9^ M^−1^ s^−1^

7 Br˙ + Br^−^ → Br_2_˙^−^*k* = 1.2 × 10^10^ M^−1^ s^−1^

8 Br˙+Br_2_˙^−^ → Br_2_ + Br^−^

9
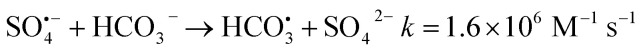

10 SO_4_˙^−^ + HO^−^ → HO˙ + SO_4_^2−^*k* = 6.5 × 10^7^ M^−1^ s^−1^

11 HO˙ + HCO_3_^−^ → CO_3_˙^−^ + H_2_O

This study aimed to investigate the degradation of FLU in UV/PDS system in different water samples including seawater, marine aquaculture water and freshwater. The effects of water quality parameters especially the concentrations of the anions in seawater on FLU degradation were tested. The transformation pathways of FLU and the oxidation products were analyzed by theoretical calculation and LC-MS/MS.^40^ The corresponding bioaccumulations of the oxidation products were also estimated by EPI-suite.^41^

## 2. Materials and methods

### 2.1. Materials and water samples

FLU (99%) was purchased from Shanghai Yuanye Biological Company. Sodium persulfate (Na_2_S_2_O_8_), sodium chloride (NaCl), sodium sulfate (Na_2_SO_4_), sodium bromide (NaBr) and sodium bicarbonate (NaHCO_3_) were analytical-reagent grade purchased from Chengdu Jinshan Chemical Reagent Company. HPLC grade formic acid, acetonitrile and methanol (CH_3_OH) were purchased from Thermo Fisher Scientific. Ultrapure water obtained from the Millipore Milli-Q Ultrapure Gradient system was used to prepare all solutions. The essential anions including Cl^−^ (19 g L^−1^), Br^−^ (65 mg L^−1^), HCO_3_^−^ (152 mg L^−1^) and SO_4_^2−^ (2.74 g L^−1^) were added into ultrapure water to prepare the synthetic seawater according the components of actual seawater. The synthetic marine aquaculture was prepared by synthetic seawater and ultrapure water in a ratio of 2 : 1. Therefore, the concentrations of the essential anions were approximately one-third of that in synthetic seawater. The pH of all samples was adjusted to 7 with phosphate buffer.

### 2.2. Experimental procedures

The UV/PDS photo-reactor was purchased from Shanghai Bilang Instruments and Equipment Company. The diameter and length of the quartz reaction tube was 2.5 cm and 18 cm, respectively. The UV light source is a high-pressure mercury lamp with a light power of 240 W. The initial concentration of FLU was set at 0.05 mM to analyze the reaction rates and products accurately. Different concentration of PDS was added and mixed with FLU. About 1 mL pure methanol was added to terminate the reaction at the set time periodically. After the reaction was quenched, the reaction solutions were collected and followed by filtration and quantitative detection. In the kinetics studies, the effects of water quality, anions and pH on the reaction were studied. When the effect of different inorganic anions on the degradation of FLU was investigated individually, the concentrations of the other three ions did not changed. All kinetic experiments were carried out in a 100 mL reaction tube.^31^ The degradation kinetic of FLU in UV/PDS system was described by a pseudo first-order kinetic model. All kinetic experiments were performed at least in duplicate.

### 2.3. Analytical methods

Ultra-high-performance liquid chromatography (UPLC, Agilent, USA) system equipped with a Zorbax RRHD Eclipse plus C18 column (2.1 mm × 100 mm, 1.8 μm), a quaternary gradient pump, a UV diode array detector and a fluorescence detector was used to determine the concentrations of FLU. The mobile phase was a mixture of formic acid (0.1%) and acetonitrile at a ratio of 70 : 30 (v/v%) with a flow rate of 0.35 mL min^−1^. FLU was detected at 234 nm.

### 2.4. Identification of the transformation products

ESI-high resolution mass spectrometry was used to identify the oxidation products. The accurate mass, isotope patterns and MS/MS patterns were obtained from a Thermo Fisher LC-MS/MS (Q-Exactive) system equipped with a Zorbax SB-C18 column (2.4 mm × 150 mm, 5 mm), a diode-array UV/vis detector and a mass spectrometer. The gradient elution was performed with a mixture of acetonitrile and 0.2% formic acid at a flow rate of 0.30 mL min^−1^. The oxidation products were identified in a positive electrospray ionization mode (ESI^+^) with a mass scan range from 50 to 1000. The Thermo Xcalibur Qual Browser and ChemSpider databases were used to infer the structures and molecular formula of the unknown products.

## 3. Results and discussion

### 3.1. Oxidation of FLU by UV/PDS in different water samples and reaction kinetics

**Fig. 1:**
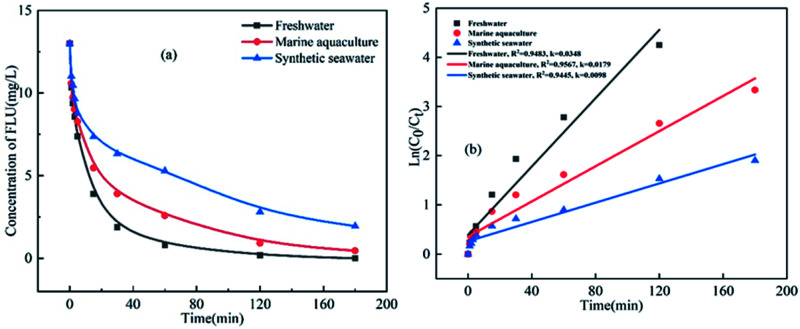
Oxidation of FLU by UV/PDS in different water samples (a) and the linear fit of the kinetic data (b) (reaction conditions: [FLU] = 0.05 mM, 50 mL, [PDS] = 20 mM, 1 mL, light power = 240 W, pH = 7, *T* = 298.15 K).

The oxidation kinetics of FLU by UV/PDS in three water samples including fresh water, synthetic marine aquaculture water and synthetic seawater were compared. As shown in Fig. 1, the concentrations of FLU decreased continuously with time at different rates. After 3 hours of reaction, FLU was almost completely degraded in fresh water. While the degradation rates of FLU in synthetic mariculture water and seawater were 96% and 85%, respectively. In the previous study, we found that FLU could not be degraded by NaClO and showed almost no reaction.^42^ Pseudo-first-order rate constants were calculated by the slopes of the fitted linear plots of ln([FLU]) *versus* reaction time *t* (Fig. 1b). It can be seen that the reaction rate constants in the three water samples were 0.0348 min^−1^, 0.0179 min^−1^ and 0.0098 min^−1^, respectively, followed the order of fresh water > synthetic mariculture water > synthetic seawater, which was contrary to the degradation law of OFL and SMX by UV/PDS in our previous research. As the main cations including sodium ions (Na^+^) and potassium ions (K^+^) in sea water were proved to have no impact on the degradation of organic compound by UV/PDS.^34^ The reason may be attributed to the high concentration of anions (*e.g.*, Cl^−^, Br^−^, HCO_3_^−^) in the mariculture water and seawater, which showed promoting or inhibiting effects on the FLU degradation by the second-generation radicals such as Cl˙, Br˙ and 
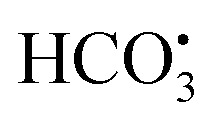
.^43–45^ Thus, the anions in the water would greatly affect the degradation of FLU.

Additionally, organic matter also has an effect on the degradation of FLU in different water samples. Qi *et al.* investigated the humic acid can affect the NP and TCS removal, as the HA concentration (>1 mg L^−1^) increased, the inhibition of NP degradation becomes enhanced.^46^ Peng *et al.* pointed out that NOMs (*e.g.*, SRHA and PLFA) adversely affected MeP removal by Co/CNTs + PMS system. The reason may be attributed to the organic matters can affect the oxidation reaction based on radicals through the scavenging effect and competing with the target contaminants for the oxidant radicals.^47^ On the other hand, organic matters might restrict the interaction of PMS with catalytic active sites. Thus, the anions and organic matters in the water would greatly affect the degradation of FLU.

### 3.2. Effects of coexisting inorganic anions on the oxidation of FLU by UV/PDS

**Fig. 2:**
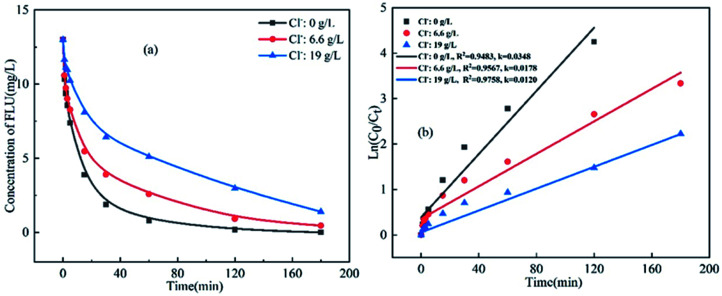
Oxidation of FLU by UV/PDS at different concentrations of Cl^−^ (a) and the linear fit of the kinetic data (b) (reaction conditions: [FLU] = 0.05 mM, 50 mL, [PDS] = 20 mM, 1 mL, light power = 240 W, pH = 7, *T* = 298.15 K).

To analyze the effects of coexisting inorganic anions on the oxidation of FLU by UV/PDS, the reaction kinetics were studied in water samples containing different amounts of inorganic anions including Cl^−^, Br^−^, HCO_3_^−^and SO_4_^2−^. The concentrations of these inorganic anions were set according to their actual concentrations in various water samples. For example, the Cl^−^ concentrations in fresh water, marine aquaculture water and seawater were 0 g L^−1^, 6.6 g L^−1^ and 19 g L^−1^, respectively. It can be seen from Fig. 2a that when the concentration of Cl^−^ increased from 0 g L^−1^ to 19 g L^−1^, the degradation efficiency of FLU decreased by 65%. The reaction rates were 0.0348 min^−1^, 0.0178 min^−1^ and 0.0120 min^−1^. The results indicated that Cl^−^ obviously inhibited the reaction and slowed down the oxidation rate of FLU. The phenomenon was consistent with the degradation of SMX, OFL and chloramphenicol by the UV/PDS system which could be attributed to the transformation of Cl^−^ in the UV/PDS system as shown in eqn (3)–(5).^27,31,33^ Therefore, the inhibitory effect of Cl^−^ on the degradation efficiency of FLU could be attributed to: Cl^−^ could capture SO_4_˙^−^ to generate Cl˙ (*E*_0_ = 2.40 V) and Cl_2_˙^−^ (*E*_0_ = 2.0 V) with oxidation properties weaker than that of SO_4_˙^−^. Moreover, the quenching reaction between Cl˙ and Cl_2_˙^−^ (eqn (5)) led to the reduction of the amounts of free radicals in the reaction system.

**Fig. 3:**
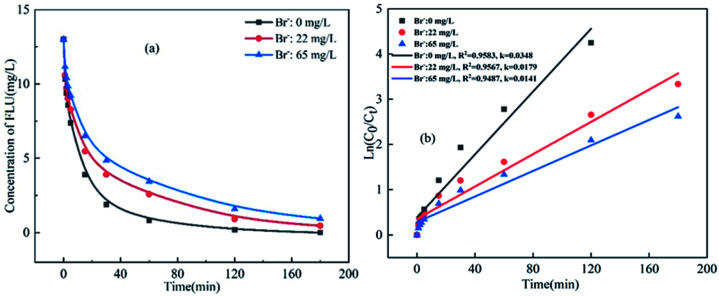
Oxidation of FLU by UV/PDS at different concentrations of Br^−^ and the linear fit of the kinetic data (reaction conditions: [FLU] = 0.05 mM, 50 mL, [PDS] = 20 mM, 1 mL, light power = 240 W, pH = 7, *T* = 298.15 K).

The effect of Br^−^ on the degradation of FLU was the same as that of Cl^−^ (Fig. 3). It can be seen that the degradation efficiency of FLU decreased gradually with the increase of Br^−^. When the concentration of Br^−^ increased from 0 mg L^−1^ (in fresh water) to 65 mg L^−1^ (in sea water), the reaction rate decreased from 0.0348 min^−1^ to 0.0141 min^−1^. The results showed that Br^−^ inhibited the degradation of FLU by UV/PDS as reported by Luca *et al.*^48^ This may be due to the fact that the transformation of Br^−^ and the properties of FLU, the new radical Br˙ (*E*_0_ = 2.00 V) and Br_2_˙^−^ were formed by Br^−^ reacted with SO_4_˙^−^, the generated radicals not only quenched each other, but also reacted with organic matters with at a slower rate than of SO_4_˙^−^.^48^

**Fig. 4:**
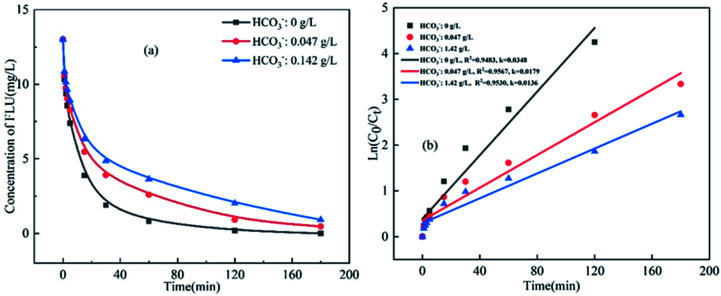
Oxidation of FLU by UV/PDS at different concentrations of HCO_3_^−^ and the linear fit of the kinetic data (reaction conditions: [FLU] = 0.05 mM, 50 mL, [PDS] = 20 mM, 1 mL, light power = 240 W, pH = 7, *T* = 298.15 K).

HCO_3_^−^ is an important component in seawater with a concentration of about 0.142 g L^−1^ and the concentration in marine aquaculture water is about 0.047 g L^−1^. As shown in Fig. 4, the degradation efficiency of FLU decreased gradually with the increase of HCO_3_^−^. In the study of Wang *et al.*,^49^ HCO_3_^−^ also inhibited the degradation of ciprofloxacin by BiFeO_3_/PDS. The reason may be due to that HCO_3_^−^ was considered as a strong free radical scavenger for both SO_4_˙^−^ and HO˙.^50,51^ The HCO_3_^−^ reacted with SO_4_˙^−^ and HO˙ (eqn (9)–(11)) to form CO_3_˙^−^ and HCO_3_˙ with weak oxidability resulting in a decrease of the total amount of free radicals in the reaction system. On the other hand, the pH value of the three water samples were different (6.6, 7.6 and 8.4 for simulated fresh water, marine aquaculture water and seawater, respectively) as HCO_3_^−^ could be hydrolyzed.

**Fig. 5:**
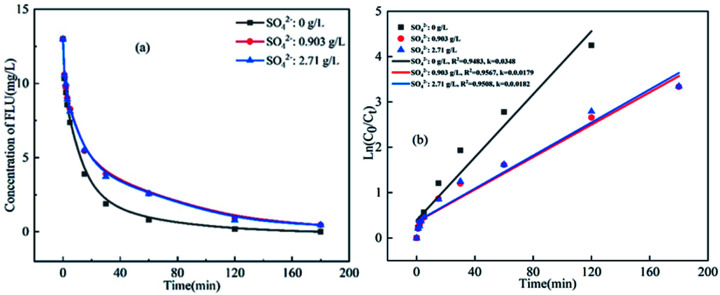
Oxidation of FLU by UV/PDS at different concentrations of SO_4_^2−^ and the linear fit of the kinetic data (reaction conditions: [FLU] = 0.05 mM, 50 mL, [PDS] = 20 mM, 1 mL, light power = 240 W, pH = 7, *T* = 298.15 K).

There is high concentration of SO_4_^2−^ in mariculture water and seawater. Thus, the influence of SO_4_^2−^ on the degradation of FLU in UV/PS was also studied. As shown in Fig. 5, the degradation efficiency of FLU was not significantly affected by SO_4_^2−^. In the different concentrations of SO_4_^2−^, the degradation rate of FLU was 96% within 180 min. When the concentration of SO_4_^2−^ increased from 0.0903 g L^−1^ to 2.71 g L^−1^, the reaction rates were 0.0179 min^−1^ and 0.0182 min^−1^, respectively. The phenomenon was consistent with the degradation of OFL.^31^ The much slower reaction of SO_4_^2−^ with radicals such as ˙OH and SO_4_˙^−^ compared to other inorganic anions (Cl^−^, Br^−^, HCO_3_^−^). Thus, the neglectable effects of SO_4_^2−^ on the degradation of FLU can be attributed to the fact that SO_4_^2−^ neither participated in the UV/PDS reaction nor changed the characteristics of the solution.

### 3.3 Effects of pH on the degradation of FLU

**Fig. 6:**
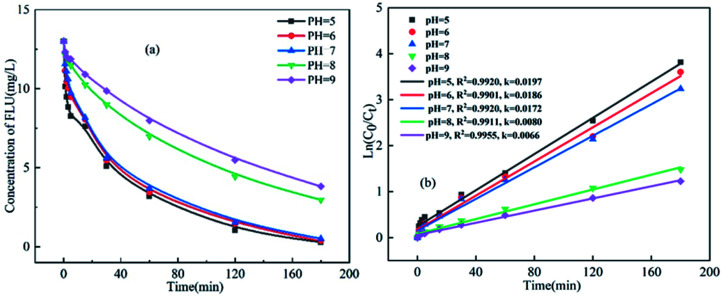
Oxidation of FLU by UV/PDS at different pH values and the linear fit of the kinetic data (reaction conditions: [FLU] = 0.05 mM, 50 mL, [PDS] = 20 mM, 1 mL, light power = 240 W, *T* = 298.15 K).

The pH value of reaction is a critical parameter influencing the progress of oxidation. Thus, the effects of different pH on the reaction rate of UV/PDS degradation of FLU were studied. Obviously, pH has a important effect on the degradation of FLU by UV/PMS. As shown in Fig. 6, the degradation efficiency of FLU decreased gradually with the increase of pH value. When the pH value increased from 5 to 9, the reaction rate decreased from 0.0197 min^−1^ to 0.0066 min^−1^. This phenomenon was consistent with the effect of HCO_3_^−^ on degradation of FLU.

The change of reaction rate with pH value may be explained from the following aspects: (1) in alkaline solution, SO_4_˙^−^ reacted with OH^−^ to produce HO˙ with a lower redox potential (eqn (10)). In addition, the coexistence of SO_4_˙^−^ and HO˙ would lead to mutual quenching which reduced the removal rate of FLU. (2) It was reported that the redox potential of SO_4_˙^−^ under acidic conditions was higher than that under alkaline conditions.^52^ (3) FLU is an amphoteric antibacterial compound, which contains amino possessing a positive charge and carboxyl groups with a negative charge. The morphology of FLU would change with different pH values of the solution. Under acidic conditions, zwitterionic functional groups would be protonated, resulting in positive charge on the piperazine ring, the electrostatic attraction between positively charged FLU^+^ and negatively charged SO_4_˙^−^ made the oxidation of FLU by UV/PDS easier. On the contrary, due to the negative charge of FLU in alkaline solution, the interaction between FLU^−^ and negatively charged SO_4_˙^−^ increased the repulsive force that resulted in the slowdown of the degradation rate. Thus, more and more FLU in protonated form were formed with the decrease of the pH, which was advantageous to the oxidation of FLU by free radicals, especially by SO_4_˙^−^.

To sum up, the increase of pH will not only change the morphology of FLU, but also affect the composition and quantity of free radicals in the solution, thus affecting the degradation rate of FLU by UV/PDS in many ways.

### 3.4 Product identification and path way analysis

**Fig. 7:**
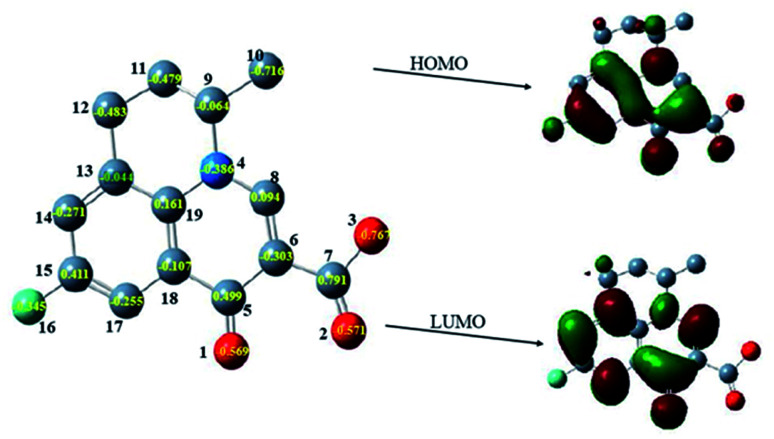
The HOMO, LUMO and net charge distribution of FLU.

The oxidation products were identified on the basis of the chemical structures and characteristics of parent compound FLU first. The density functional theory (DFT) has been widely used as it can obtain the physicochemical properties of compounds to reveal their changing tendencies.^53^ A large number of researchers have used DFT to calculate and predict the environmental behavior of organic pollutants, as well as conduct risk assessment on the toxicity of transformation products.^54^ The quantum chemical parameters including the frontier orbital characteristics and charge distribution of FLU were calculated by Gaussian09 program according to DFT. As it was difficult to extract electrons from a low-lying highest occupied molecular orbital (HOMO) and add electrons to a high-lying lowest unoccupied molecular orbital (LUMO), the degradation potential of FLU was preliminarily analyzed by the difference calculation of quantum chemical parameters between *E*_HOMO_ and *E*_LUMO_ (Δ*E*). A large value of Δ*E* indicated a high kinetic stability and low chemical reactivity.^55^ Δ*E* was calculated to be 8.07 eV which was much higher than that of other fluoroquinolones indicating that FLU is very stable. The main reaction active sites on FLU molecules were determined according to the net charge distribution by natural bond orbital analysis. As shown in Fig. 7, the electrons of HOMO and LUMO were mainly distributed on quinolone group indicating that the quinolone ring would easy to be attacked not only by electrophilic reagent but also nucleophilic reagent. The charge values of the atoms C6, O1, O2, O3 and N4 on the quinolone group were calculated to be −0.386 eV, −0.569 eV, −0.571 eV, −0.767 eV and −0.303 eV, respectively. These atoms with high negative charge would be more vulnerable to attack by free radicals through electrophilic reactions.

**Fig. 8:**
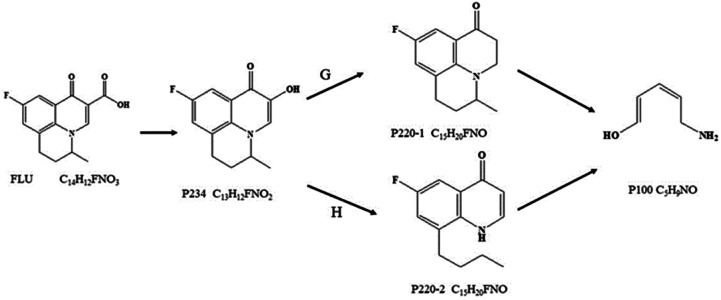
Possible degradation pathway of FLU by UV/PDS system in freshwater, marine aquaculture water and seawater.

The transformation products were identified by the accurate masses obtained from LC-MS/MS combined with the reaction active sites analysis. One final product and three intermediate products were identified, while no halogenated compound was found in marine aquaculture water and seawater. Based on the above transformation products and reaction sites analysis, the degradation of FLU was inferred including two possible pathways G and H (Fig. 8). Both the two pathways had the same intermediate product p234 (*M*/*Z* = 234) which was formed by the oxidation of carboxyl (–COOH) of FLU to hydroxyl group (–OH). Through the pathway G, hydroxyl group (–OH) of p234 was oxidized to form p220-1 (*M*/*Z* = 200). In pathway H, the C–N bond of p234 were attacked and broken to form p220-2 (M/Z = 200). And then the quinolone groups on P220-1 and p220-2 were oxidized to form the final product P100 (*M*/*Z* = 100). The reaction products and pathways were the same in the three water bodies.

### 3.5 Preliminary analysis of bioaccumulations

The bioaccumulation characteristics of the degradation products were analyzed by Epi suite software. Log *K*_ow_ and log BCF were selected as the representative indexes. The compound with greater values of Log *K*_ow_ and log BCF indicated that it would be more easily absorbed by cellular organisms and dissolved in nonpolar media. The calculation results were shown in Table 1. The log *K*_ow_ and log BCF of FLU was 1.60 and 0.579, respectively. Log *K*_ow_ of the three intermediates p234, p220-1 and p220-2 was 2.50, 3.28 and 3.51, respectively. Log BCF of the three intermediates was 0.731, 1.248 and 1.401, respectively. Log *K*_ow_ and log BCF of the final degradation product P100 were both less than 0. The results showed that the values of Log *K*_ow_ and log BCF of the three intermediates were greater than those of FLU, indicating that compared with FLU, the three intermediates were easier to accumulate and more toxic in organisms. The values of log *K*_ow_ and log BCF of the final product P100 were less than those of FLU and the intermediates, indicating that P100 exhibited weak bioaccumulation and toxicity. The above results suggested that UV/PDS is suitable for the application of FLU degradation in fresh water, marine aquaculture water, synthetic seawater.

**Table 1:** Bioaccumulations of FLU and the oxidation products

Compound	Structure	Log *K*_ow_	Log BCF
FLU C_14_H_12_FNO_3_	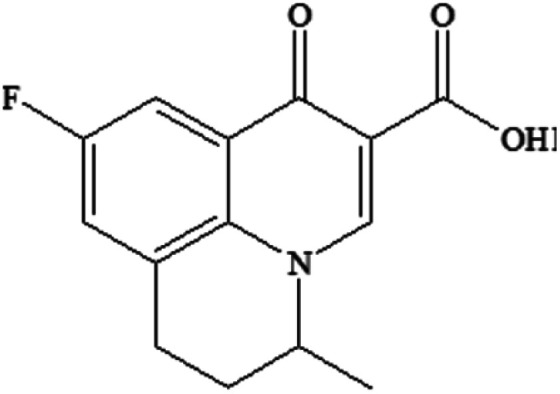	1.60	0.579
P234 C_13_H_12_FNO_2_	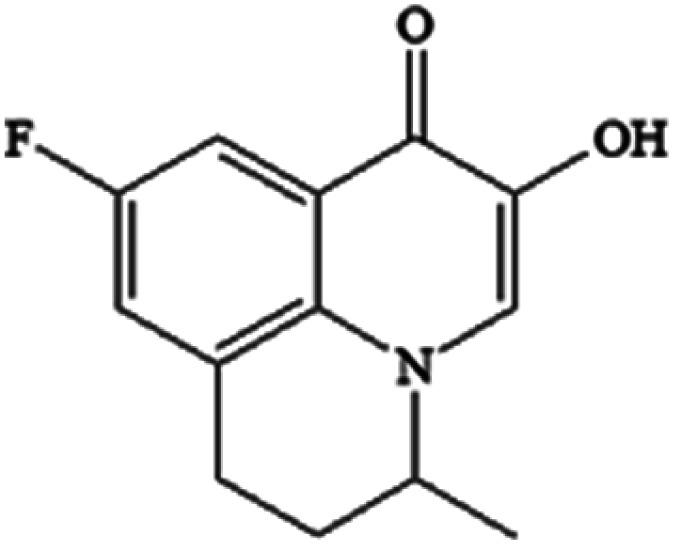	2.50	0.731
P220-1 C_15_H_20_FNO	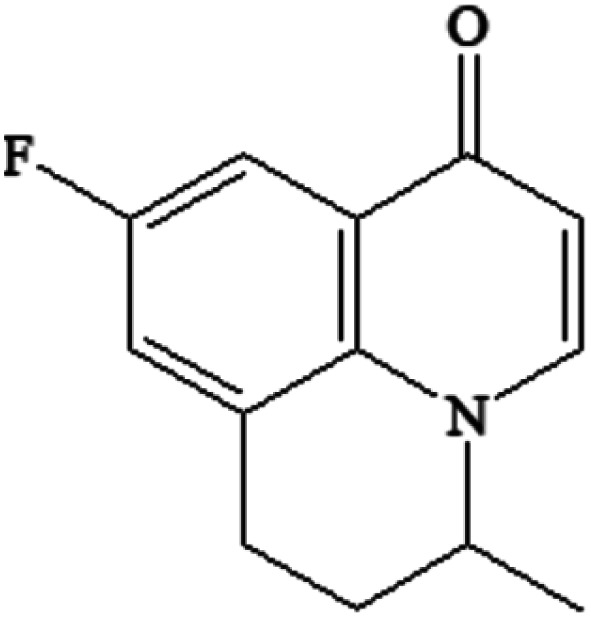	3.28	1.248
P220-2 C_15_H_20_FNO	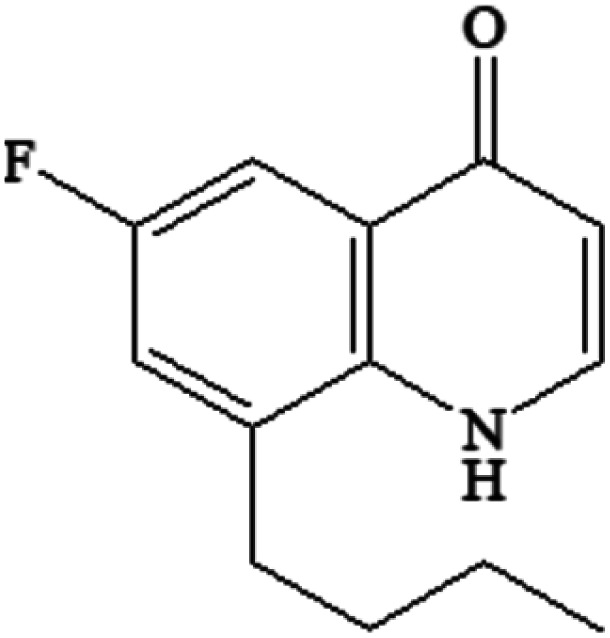	3.51	1.401
P100 C_5_H_9_NO	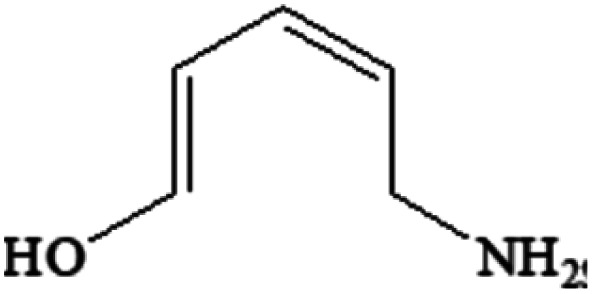	<0	<0

## 4. Conclusions

As a member of FQs group, FLU was degraded by UV/PDS in synthetic freshwater, marine aquaculture water and seawater with different amounts of inorganic anions. The results showed that the reaction rate constants of FLU in the three water samples followed the order of fresh water > marine aquaculture water > synthetic seawater. The anions with higher concentrations in seawater including Cl^−^, Br^−^ and HCO_3_^−^ inhibited the degradation of FLU. The reason should be partly attributed to that the anions could capture SO_4_˙^−^ to generate new reactive radicals such as Cl˙, Br˙ and CO_3_˙^−^ with oxidation properties weaker than that of SO_4_˙^−^. The degradation efficiency of FLU was neglectable affected by SO_4_^2−^, which can be attributed to the fact that SO_4_^2−^ neither participated in the UV/PDS reaction nor changed the pH of the solution. The degradation efficiency of FLU decreased gradually with the increase of pH value. The quenching reaction of free radicals, the difference of redox potential of SO_4_˙^−^ and the morphology of FLU under different pH values should be responsible for the effect of pH on the degradation of FLU. The main reaction active sites on FLU molecules were the atoms C6, O1, O2, O3 and N4 on the quinolone group. Three intermediate products with greater values of log *K*_ow_ and log BCF were identified. The final product P100 exhibited weak bioaccumulation.

## Conflicts of interest

There are no conflicts to declare.

## Supplementary Material

RA-012-D2RA00199C-s001
