# Supplementary material for: Kinetics and mechanisms of flumequine degradation by sulfate radical based AOP in different water samples containing inorganic anions
Source: RSC Adv. 2022 Mar 30;12(16):10088–96. doi: 10.1039/d2ra00199c (PMC8966717; doi:10.1039/d2ra00199c)
Supplement: RA-012-D2RA00199C-s001 [file RA-012-D2RA00199C-s001.pdf]

Electronic Supplementary Material (ESI) for RSC Advances.
This journal is © The Royal Society of Chemistry 2022

Supplementary Data

Kinetics and mechanisms of flumequine degradation by sulfate radical based AOP in different water samples containing inorganic anions

Yuanyuan Zhang [1,2] , Kunling Huang [1] , Yunjie Zhu [1] , Xuan Chen [1] , Min Wei [1] , Kefu Yu [1*,2]

[a] School of Marine Sciences, Guangxi Laboratory on the Study of Coral Reefs in the South China Sea, Guangxi University, Nanning 530004, China

[b] Southern Marine Science and Engineering Guangdong Laboratory (Zhuhai), Zhuhai 519000, China

List of Supporting Figures

Figure. S1. LC/MS-ESI$^+$ spectra for FLU.

Figure. S2. LC/MS-ESI$^+$ spectra for intermediate product (P234) of the reaction between FLU.

Figure. S3. LC/MS-ESI$^+$ spectra for intermediate products (P220-1 and P220-2) of the reaction between FLU and UV/PDS.

Figure. S4. LC/MS-ESI$^+$ spectra for final product of the reaction between FLU and UV/PDS.

Figure. S1.

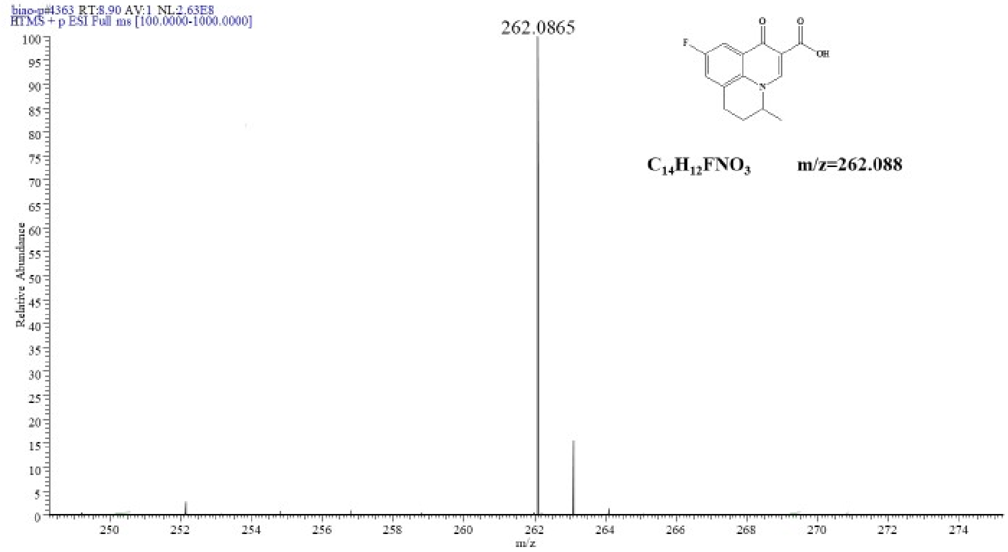

Figure. S2.

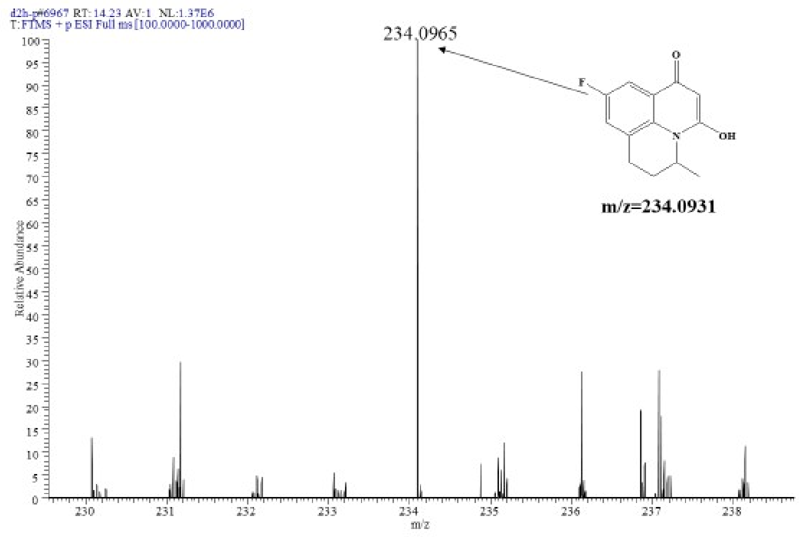

Figure. S3.

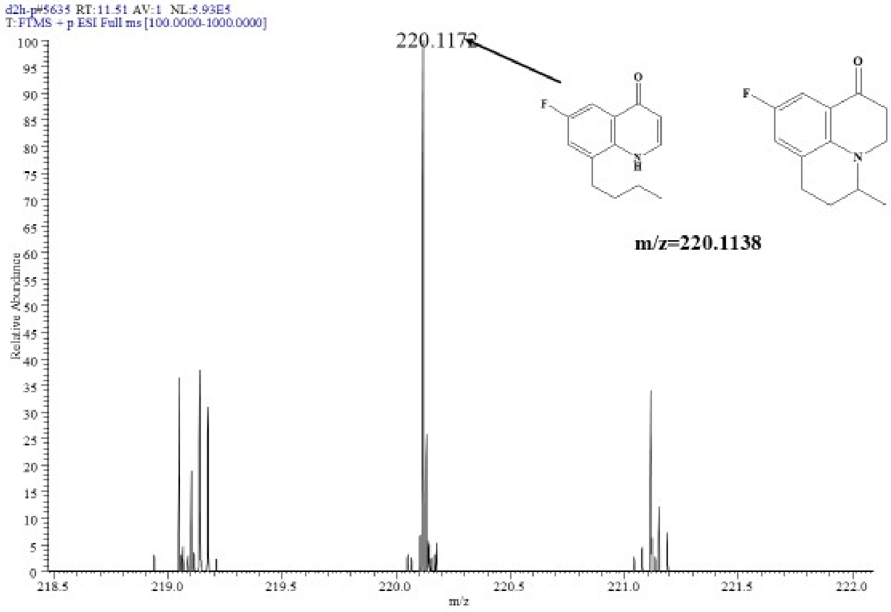
d2h-p#5635 RT:11.51 AV:1 NL:5.93E5
T: FTMS + p ESI Full ms [100.0000-1000.0000]
220.1172
m/z=220.1138
Relative Abundance
m/z

Figure. S4.

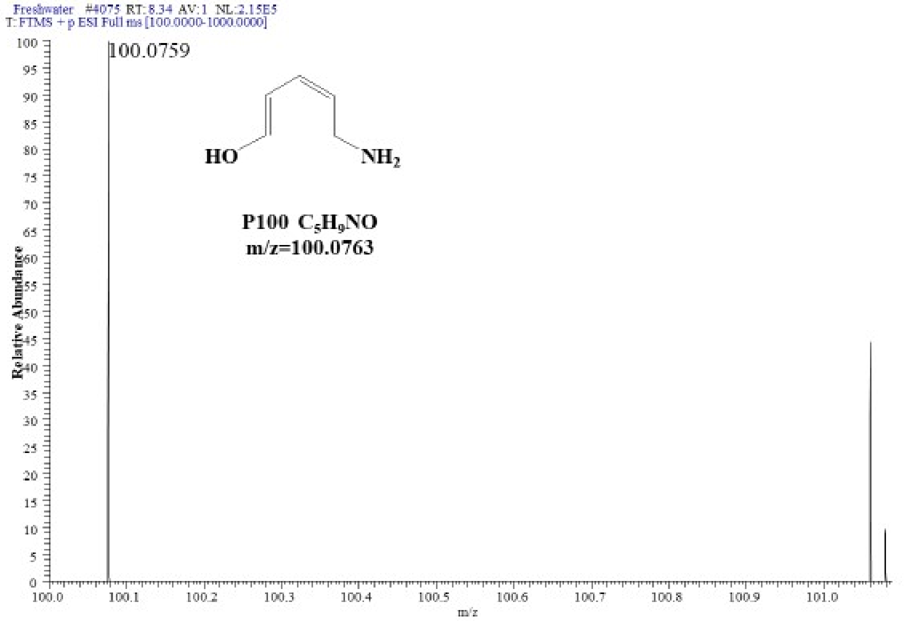
Freshwater #4075 RT:8.34 AV:1 NL:2.15E5
T: FTMS + p ESI Full ms [100.0000-1000.0000]
100.0759
HO
NH2
P100 C5H9NO
m/z=100.0763
Relative Abundance
m/z
